# Microfluidics Mediated Production of Foams for Biomedical Applications

**DOI:** 10.3390/mi11010083

**Published:** 2020-01-12

**Authors:** Ilham Maimouni, Cesare M. Cejas, Janine Cossy, Patrick Tabeling, Maria Russo

**Affiliations:** 1 Microfluidics, MEMS, Nanostructures Laboratory, CNRS Chimie Biologie Innovation (CBI) UMR 8231, Institut Pierre Gilles de Gennes (IPGG), ESPCI Paris, PSL Research University, 6 rue Jean Calvin, 75005 Paris, France; maimouni.ilham@gmail.com (I.M.); cesare.cejas@gmail.com (C.M.C.); patrick.tabeling@espci.psl.eu (P.T.); 2 Molecular, Macromolecular Chemistry and Materials, ESPCI Paris, CNRS, PSL University, 10 Rue Vauquelin, 75231 Paris, CEDEX 5, France; janine.cossy@espci.psl.eu

**Keywords:** microfluidics, foams, polymer foams, tissue engineering, biomedical, scaffolds

## Abstract

Within the last decade, there has been increasing interest in liquid and solid foams for several industrial uses. In the biomedical field, liquid foams can be used as delivery systems for dermatological treatments, for example, whereas solid foams are frequently used as scaffolds for tissue engineering and drug screening. Most of the foam functionalities are largely correlated to their mechanical properties and their structure, especially bubble/pore size, shape, and interconnectivity. However, the majority of conventional foaming fabrication techniques lack pore size control which can induce important inhomogeneities in the foams and subsequently decrease their performance. In this perspective, new advanced technologies have been introduced, such as microfluidics, which offers a highly controlled production, allowing for design customization of both liquid foams and solid foams obtained through liquid-templating. This short review explores both the fabrication and the characterization of foams, with a focus on solid polymer foams, and sheds the light on how microfluidics can overcome some existing limitations, playing a crucial role in their production for biomedical applications, especially as scaffolds in tissue engineering.

## 1. Introduction

Foams are lightweight materials made from dispersions of gas bubbles in a continuous matrix which can either be liquid or solid, giving birth to liquid or solid foams respectively [1]. Foams can find a variety of applications in different industrial sectors [2] depending on their type. Liquid foams are widely used in detergents [3,4,5], food [6], cosmetics [7,8,9,10], fire-fighting [11], oil recovery [12], and pharmaceutical applications [7,13,14,15,16,17,18]. Solid foams can find applications as insulation materials [19] and as packing and cushioning materials [20]. In general, various polymers are used in solid foams: polyurethane (PU), polystyrene (PS), polyethylene (PE), polypropylene (PP), poly(vinyl chloride) (PVC), polycarbonate (PC), etc. Recently, interest in solid polymer foams has soared for biomedical applications [21] as scaffolds thanks in large part to their tunability of pore-network control and mechanical properties. Some of these applications include tissue engineering [22,23], tissue regeneration, cell culture, drug delivery [7,13,14,15,16,17,18,24], bio-sensing, and diagnostics [25]. To design biomedical foams which are biocompatible and biodegradable, biologically or synthetically-derived biomaterials [26] are privileged, especially for soft tissue applications [27,28,29] due to their organic matrix. However, metals [1,23] and ceramics [30] can also be employed for the fabrication of medical devices for hard tissues, such as artificial prostheses [31], bone scaffolds [32], and dental implants [33]. Furthermore, composites [34] (by combining two or more materials) are also used in order to take advantage of specific characteristics of the individual components.

Different conventional and advanced processing techniques have been developed to fabricate liquid and solid foams. In the biomedical field, the choice of a specific fabrication method is critical to get biomedically-engineered foams with customized properties. In fact, one of the paramount parameters of biomedical foams is the tuning of porosity that depends on the processing technique. Generally, conventional fabrication methods of liquid foams include bubbling into stationary liquids, co injection of gas and liquid, nucleation, and growth of bubbles [35]. All these techniques can lead to solid foams through liquid-templating [36]. Additionally, solvent casting/particle leaching (SCPL), thermally-induced phase separation, freeze-drying, gas foaming, and melt moulding [27,28] are commonly used for solid foams fabrication. For these methods, porosity is varied by tuning different parameters such as temperature, liquids’ viscosities and reactants’ concentrations. However, solid foams obtained by using these techniques tend to have pores with wide distributions of size and shape. The inhomogeneity in porous texture has caused these foams to be much less successful than their potential suggests. When such structures are used as scaffolds in tissue engineering, previous studies reveal that high variability of the pore size creates impediments to cell seeding and growth [37]. Furthermore, a uniform porous texture guarantees a more homogeneous distribution of cells and an even mass transfer in all compartments of the scaffold [38]. The fine-tuning of structures, and thus their properties by liquid templating routes, is an emerging field, which deserves to be explored [29]. Advanced structural requirements demand a manufacturing technology capable of systematically tuning 3D scaffolds. The recent progress of technology has allowed the development of advanced fabrication methods of scaffolds, such as electrospinning [39], 3D printing [40], and rapid-prototyping [41]. In parallel, the emergence of microfluidics offered a new way to produce scaffolds, increasing the achievable resolution by modulating self-assembled liquid foams’ architectures and compositions, thereby improving their performances [12,42,43,44,45]. From that perspective, microfluidics, by handling small quantities of fluids, can generate a monodisperse collection of bubbles that can be scaled down to the length of a few microns, and potentially even lower [46]. Scaffolds are obtained through solidification of these preformed liquid foams [36]. The ability to control biomaterial physico-chemical properties down to micrometer and nanometer scales [47] has opened new routes in biomedicine for cell transplantation [48], drug release [9,49], health monitoring [50,51,52], diagnostics [53], and therapeutic treatments *in situ* [54,55].

This short contribution reviews the principal batch fabrication methods used to produce liquid and solid foams, showing both their advantages and disadvantages, and then how the advent of microfluidic approaches can overcome some limitations and bottlenecks (of batch processes) related to the production. In relation to this, the most common techniques to fabricate microfluidic devices and relevant microfluidic geometries for designing foams, liquid and solid respectively, are then described. Later, a special section is dedicated to the characterization tools of the so-far described structures. Finally, we present how microfluidics can play a crucial role in foam production for biomedical applications, particularly for scaffolds in tissue engineering, due to the strong dependence of their biomechanical properties on pore size, porosity, and geometry, which can all be easily tuned by microfluidics. We focused this short review on polymeric foams because, thus far, these are the types that can be handled with microfluidics.

## 2. Foam Fabrication Techniques

Generally, the generation of a liquid foam is not spontaneous but requires the input of an energy to create the gas–liquid interface with a defined surface tension. Depending on how this energy is brought to the system, the foaming method is either biological, chemical, or physical [35]. By adding surface-active components, such as surfactants, to reduce the surface tension and create bubbles interfaces, physical methods generally require the application of a mechanical force (shaking) at the fluid or phase transitions of dissolved gases’ (nucleation and growth [56], cavitation [57]). Chemical foaming generally occurs due to a gas-releasing chemical reaction [58], while biological methods rely most often on gas-generating species, including yeast and bacteria (proteins influencing foam formation in wine and beer: the role of yeast). Drenckhan et al. reported an exhaustive review about the most common techniques of liquid foam formation, including bubbling into a stationary liquid, co-injection of gas and liquid, bubble breakup under shear, etc. [35]

The methods described so far allow one to generate liquid foams, and solid foams (such as scaffolds) as well, through liquid-templating, for example [36]. For such a perspective, physical methods are considered to be the best in terms of the control allowed over the characteristics of the foams [59]. In the following, we review most common conventional and advanced physical fabrication techniques of polymer solid foams, highlighting their advantages and their limitations [27,28]. Conventional techniques include:

**Gas foaming**: A porous structure is created through the nucleation and growth of gas bubbles dispersed throughout a polymer phase. Solid discs of a scaffold material are created using compression molding. Then, the discs are saturated with carbon dioxide (CO${}_{2}$) by exposing them to high pressure of CO${}_{2}$ gas for 72 h at room temperature before the solubility of the gas in the polymer gets rapidly decreased by reducing CO${}_{2}$ pressure to atmospheric levels (P${}_{0}$ CO${}_{2}$). This causes the CO${}_{2}$ gas to clump together, creating pores. High porosities (up to 93%) and pore size (up to 100 mm) can be obtained, but the control over the connectivity and the pore size is not straightforward [60]. Moreover, the high pressure required for the process is not appropriate for some bio-applications (for example, as it may be incompatible with inclusion of cells and bioactive molecules directly into scaffolds) [32].

**Solvent casting/particulate leaching**: This is one of the most widely used methods for foam generation. It involves the use of a polymer solution mixed with salt particles (generally NaCl) of a defined size. When the solvent evaporates, it leaves behind a porous polymer matrix. This technique presents the advantage of being relatively simple and allowing the creation of scaffolds with regular porosity, and controlled composition and pore size—though the control of the pore shape and interconnectivity is not guaranteed, and the achievable mechanical properties and material dimensions remain limited. Furthermore, the use of organic solvents limits the potential bio-applications [61,62].

**Thermally induced phase separation (TIPS)**: TIPS [63] is based on the change in temperature to induce the de-mixing of a homogeneous polymer solution, thereby creating a multi-phase system. The solution is quenched, producing a liquid–liquid phase separation—a polymer-rich phase and a polymer-poor phase. The de-mixing can be solid–liquid (usually for binary polymer-solvent mixtures), or liquid–liquid (usually for ternary polymer/solvent/non-solvent mixtures). The polymer-rich phase solidifies and the polymer-poor phase crystallizes. The crystals are removed, leaving a highly porous structure (more than 90%). The advantage of the phase separation technique is that the morphology of the scaffold can be controlled by tuning parameters, such as the polymer type and concentration, the freezing temperature, and the types of porogens [63,64].

**Melt molding**: Melt molding consists of heating of polymers above their glass transition temperature or melting point, so they can assume a liquid form. The resulting polymer is then allowed to cool and solidifies in the form of the mold. After, particle leaching is used to introduce porosity into the scaffold. This technique avoids the use of polymer solvents, opening the way for non-toxic materials demanding applications. The melt molding is perfectly scalable, which makes it very advantageous for industrial applications, though it is challenging to leave non-porous layers on the surface of the final polymer and some porogen residuals in the scaffold, due to the difficulty of leaching out the particles [65,66].

**Emulsion/freeze-drying**: This method requires the formation of an emulsion first by dissolving a polymer in a solvent, and then mixing with water to form a water/oil emulsion. Then, the mixture is poured unto a mold and frozen before the two phases can separate. The frozen emulsion is then dried to remove the solvent and the dispersed water, creating pores in a solidified scaffold. Thus, these obtained pores are highly interconnected, which is appropriate for nutrient supply, metabolic waste clearance, cellular in-growth, and vascularization in biomedical applications. Though this technique does not require the use of solid porogens, organic solvents are used. The processing time can be long, the porosity is often irregular, and the pore size is limited [67,68].

All the conventional methods discussed until now offer limited control over pore size, geometry, and interconnectivity, even if large ranges can be achieved. These parameters are usually crucial for the efficiency of the scaffolds in many applications. Several studies demonstrated that combinations of conventional techniques may improve control over final structures [69,70,71]. For example, Guarino et al. [61] showed how combinations of particulate leaching, phase separation, and gas foaming may represent a way to obtain porous structures with tailored properties. Alternatively, additive manufacturing technologies offer a pathway to achieving competitive scaffold quality [40], even though serious bottlenecks are slowness, cost, and, in some cases, insufficient resolution.

In this context, the most promising techniques in terms of control over the structure and biocompatibility are, so far, physical means, especially those relying on bubbles forming via hydrodynamic instabilities such as microfluidics. This technique can offer a tool to study the relationship between the porous texture and several scaffold properties, for instance, the mechanical, the insulation, and the acoustic properties. Furthermore, in the case of cell culture, microfluidics allows correlating the influence of pore size or inter-connectivity on the different biological responses of seeded cells.

## 3. The Use of Microfluidics for Production of Foams

### 3.1. Bubble Production Using Commonly-Used Microfluidic Geometries

Using microfluidic technologies, a high-throughput production of bubbles can be generated with controlled dispersity [43] through an interplay of gas flow rate $Q_{g}$ and the liquid flow rate $Q_{l}$. The transition from isolated bubbles to foam structures can be achieved by increasing the volume fraction of the bubbles with respect to the continuous liquid phase. Using different microchannel geometries, one can control the design and frequency of production as the gas phase passes through an orifice or a junction [72,73] at the intersection of an upstream liquid pressure [43]. The geometry of the junction, together with the flow rates, channel wettability, and the physical properties of the fluids (e.g., surfactant concentration, interfacial tension, and viscosity), determine the local flow field, which deforms the interface and eventually leads to drop or bubble pinch off [74]. The pinch-off or break-up is a result of an instability at the junction of two immiscible fluids. Capillary instabilities are also stabilized by the presence of walls, and thus, under confinement, the bubble generation becomes an interplay of capillary forces and viscous friction [43,75]. The diameter of the bubble or droplet is set by a competition between the pressure due to the external flow and viscous shear stresses on the one hand, and capillary pressure resisting deformation on the other hand [74]. As a result, capillary numbers, $Ca=\frac{\eta U}{\gamma }$, are typically small ($Ca<10^{-2}$) [43,76], where γ is the interfacial tension, η is the dynamic viscosity of the fluid, and *U* is the velocity of the flow. The lubrication film between the bubble and the wall also helps in the comprehension of bubble velocity as it moves through the confinement [43,77]. The fine-tuning and comprehension of all these subtle parameters can produce foams of a controlled size, shape, and interconnectivity, and even improve production rate. A detailed review on bubble mechanisms in microfluidics can be found in [35,43].

**Figure 1:**
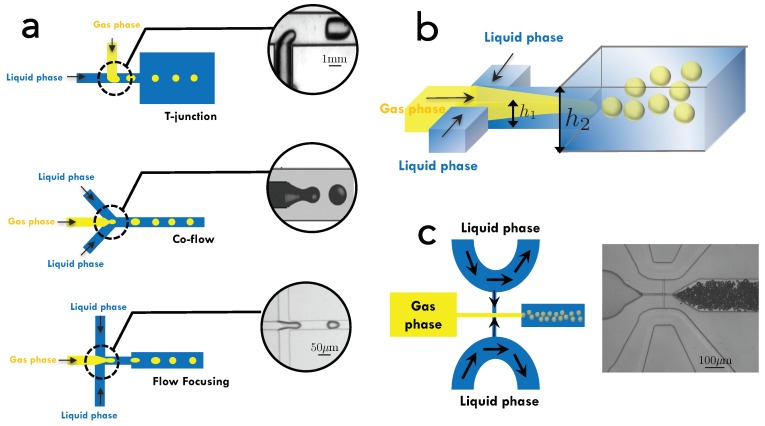
(**a**) Geometries used in the production of foams using microfluidics. In the case of flow focusing, the liquid streams focus the gas jet through a tiny orifice. For a T-junction, the vertical branch of the “T” stands for gas inlet, while the horizontal branch stands for the liquid stream and forming bubbles or droplets when they meet. Inset photo from [90]. For co-flow, both phases flow along the gradients of pressure—and in confinement by the walls of the devices. In all cases, bubble formation is obtained thanks to the periodic pinch-off of the gas jet by the liquid stream. Photo reproduced with the permission from [91]. (**b**) Illustration of a step-emulsification device where the stream meets a step change in the height of the microchannel (from $h_{1}$ to $h_{2}$). (**c**) An example of a microfluidic step emulsification device (MSE) with U-turn microchannels to prevent clogging of the narrow nanochannels due to unwanted dust particles.

Microfluidic devices for foam production can be fabricated using standard techniques such as soft photolithography [46], using a flexible silicon material (e.g., polydimethylsiloxane—PDMS), micro-milling fabrication [78] using thermoplastics (e.g., polymethyl methacrylate—PMMA), and a hybrid technique combining micromilling and gelatin-based replica molding [79]. Recently, novel protocols were developed to fabricate reversibly bonded microfluidic chips [80,81], which simplify the recovery and characterization of the sample. Circular glass capillary tubes have also been employed in some reported examples [82]. The most commonly used microfluidic geometries to generate bubbles for foam structures, shown in Figure 1a, include flow-focusing, T-junctions, and co-flowing junctions. These designs allow the production of bubbles with highly-controlled sizes ranging from few micrometers to millimeter sizes and with polydispersity lower than 5%. As these geometries are fairly common, their characteristics and flow dynamics have been widely discussed in literature [43]. For instance, using a flow-focusing technique made of capillary tubes, the diameter of the resulting bubble, $D_{b}$, scales with the capillary diameter, $D_{c}$, and the ratio $Q_{g}/Q_{l}$, which also sets the gas fraction and rate of production [82]:

(1) $$D_{b}\approx D_{c}{\left(\frac{Q_{g}}{Q_{l}}\right)}^{1/3}.$$

The foam final properties can be controlled by the dimension of the capillary (or channel) and the corresponding flow rates. In microfluidics, the high control over flows of liquids of normal viscosities comes from the fact that flow, at this scale, is dominated by viscous forces over inertia. Due to the small dimensions of micro-channels, the Reynolds Number, $Re=\frac{U\rho l}{\eta }$, where ρ is the density of the fluid, η is the dynamic viscosity of the fluid, *U* is the velocity of the flow, and *l* the characteristic dimension of the system) is usually Re<<100, often Re<1 [43]. In this regime, flow is completely laminar and no turbulence occurs. For flow in microfluidic channels, the transition to turbulent flow generally occurs in the range of Re∼2000 [83]. Laminar flow provides a means by which fluid can be transported in a relatively predictable manner through micro-channels where streamlines of the fluids can be controlled by an appropriate design of the geometry of the channels. However, even at Re<100, it is possible to have momentum-based phenomena such as flow separation.

There is another type of geometry that has been gaining exposure. That is the microfluidic step emulsifier (MSE) [84,85] mainly used for generating bubbles of smaller sizes, d<5 μm, as shown in Figure 1b. This is a step emulsification device [84,85,86], where a shallow channel with two co-flowing immiscible fluids goes into a step change in the microchannel height [84,86]. This sudden change in height allows the stream of the dispersed phase to break into droplets. This is because as the two immiscible fluids flow side by side, they are separated by a meniscus that induces a pressure difference. However, when arriving at the step height, the two fluids are forced to balance out their pressures, leading the dispersed phase to move faster while the continuous phase moves more slowly. This mass conservation forces the fluid boundary to adopt a tongue-like shape [85] that eventually thins out. A variation of the MSE device contains practical U-turn microchannels (or “rivers”) [85] as microchannel inlets, as shown in Figure 1c. A small fraction of the fluid imposed in this inlet flows into the nanofluidic section, all the while preventing the risks of clogging [87,88] from unwanted dust or other larger particles [89]. Through an MSE device, the fabrication of droplets or bubbles with d∼1 μm has been reported [85]. However, generating foam structures of sub-micrometric or nanometric pore sizes still remains the next frontier in microfluidic foam production. Nevertheless, most foam pore sizes currently used in biomedical applications are in the order of the size of a typical biological cell (∼μm range). In the future, if one wants to create biomedical foams that resemble the structure of an extracellular matrix, the challenged then is to conceive foams with nanometric pore sizes.

In general, the performances of these microfluidic devices with different geometries depend on the equilibrium of interfacial, viscous, and inertial forces during bubble formation. Each geometry has a certain range of flow rate ratios in which it is possible to obtain monodisperse foams. In most cases, the foaming solutions, used for the generation of solid porous polymer foams [29], are highly viscous or have non-Newtonian flow properties. The rheological properties of the liquid phase, in the case of a polymer liquid phase, can affect final bubble diameters. Among the characteristics to be considered are polymer molecular weight, polymer concentration, biopolymer intermolecular interactions, and polymer-surfactant interactions [92].

### 3.2. Formation of Liquid Foam Structures from Bubbles

When there is a high density of bubbles, they come into contact and nucleation occurs, thereby forming foams in the micro-channel outlet. There are principal mechanisms involved in the formation of foams, such as Ostwald ripening, drainage [45], and coalescence [72,93]. Describing these mechanisms in detail is not the subject of the present paper, but can easily be found in standard literature [43,73] and textbooks on foams [72]. In the majority of the cases where sizes are monodisperse, bubbles promptly self-assemble in an ordered structure, whose arrangement depends on the flow. Foam structures and various lattices, from circular bubbles to hexagonally close-packed structures, can easily be tuned by increasing the gas pressure [73]. Contrary to colloidal structures, gas bubbles do not require an additional energy input for self-organization due to the absence of solid friction [82]. Foams can also rearrange, thereby producing intermediate transition regimes that contain a mixture of structures [73,76]. Aqueous foams, where gas bubbles are dispersed in an aqueous liquid phase, are the most common types of liquid foams, although non-aqueous foams also exist, which are composed of gas bubbles dispersed in their respective non-aqueous solvents [94]. Non-aqueous foams need different types of foam stabilizers and it is the interaction between the stabilizer and the liquid phase that determines its properties.

Generally, foams destabilize due to the interplay of different aging phenomena, such as the rupture of the films separating the bubbles (coalescence) [44,93], the decrease of the liquid volume fraction under gravity (drainage) [45], and gas transfer between bubbles (coarsening) [95]. In the case of coalescence, for instance, it has been recently reported that the coalescence probability is largely stochastic [93]. These destabilization effects can happen simultaneously, enhancing one another and leading to the breaking of the foam structure [96]. To avoid these mechanisms, the use of stabilizing agents is necessary. Usually, different chemical surfactants (such as SDS [3,4]) are used, but polymers (e.g., partially-hydrolyzed polyacrylamide for EOR applications [5]), proteins, or particles (e.g., biopolymer-based particles [97]) can also be useful for some specific applications.

### 3.3. Formation of Solid Foam Structures

Solid foams are self-assembled porous structures, which are employed as scaffolds with varying pore morphologies that cater to their clinical application [98,99] in tissue engineering. The solid porous structure mimics the properties and functions of the extracellular matrix (ECM) by providing structural support for cells to attach, grow, and differentiate [22]. Control of scaffold pore sizes, monodispersity, and interconnectivity are crucial, since they directly influence cell seeding distribution efficiency [100] and growth [99]. The conventional method for the fabrication of foam scaffolds is gas foaming [61,101], but that methods cannot provide strict tuning of different features and functionalities of the foams, which are uniformity, pore size control, and interconnection. In the last decade, the advent of 3D printing has been also recently used to “print” or construct porous scaffolds [102], and in general this requires understanding of specific visco-elastic properties. Such techniques are based on algorithms and do not give a direct self-assembly compatible fabrication method, increasing production time and cost. In general, these techniques have been used not just for biomedical applications but also for thermal insulation applications. While generating bubbles is a common strategy for fabricating liquid foams, they can also be used as an essential starting point in the fabrication of solid foams using a method called liquid foam templating [36]. Although a number of conventional methods exist for producing solid foams (e.g., melt molding [65,66] and gas foaming [61,101]), liquid foam templating is one example that can be mediated with microfluidics. This is because its scaffold structure is built on the formation of liquid foams [36,38,59], whose connectivity and bubble size can be carefully controlled with microfluidics up to 800 μm [103], as has been widely reported in the literature [38,100,103,104,105,106,107,108,109].

The initial formation begins with standard microfluidic techniques, often flow-focusing, where two immiscible fluids meet at a defined orifice junction to generate bubbles exactly in the same manner as the formation of liquid foams. The only difference is that in the latter, the foam structure (formed from bubble nucleation) is left in its liquid state, while in the former, precursors (e.g., initiators or cross-linkers) are mixed within the continuous phase. These are used to solidify the liquid foam once the foam has been permitted to find its equilibrium structure. In this regard, as the name of liquid foam templating suggests, the initial liquid foam indeed serves as a template or a mold for the eventual solidification. The fluids used in this technique are evidently monomers in their liquid state, which polymerizes into their solid form. These polymeric foams are often referred to as cellular solids with tunable mechanical and even thermal properties [110]. One example is the synthesis of polystyrene foams from the polymerization of styrene monomer emulsions generated from microfluidic flow-focusing [111]. However, polystyrene foams are mainly generated for insulation and packing applications. Oil-based materials are fairly uncommon in biomedical applications due to their hydrophobic nature, although recent studies have reported the use of oil-in-water emulsions as cell carriers for tissue engineering [105]. Another example of the use of liquid foam templating is the fabrication of monodisperse chitosan foams or chitosan/cellulose nanocomposites [107]; more examples include alginate-based foams [100,112]. Chitosan and aliginate are both biocompatible, and thus their foams have potential tissue engineering applications. Solidfication of the liquid foam template involves heating [111], freeze-drying [113,114], and/or cross-linking [103] through the presence of photoinitiators, as in the case of gelatin methacryloyl (GM) foams [115], where generated liquid foams from microfluidic bubbling is exposed to UV light. Recent developments have also employed valve-based microfluidic flow-focusing (vFF), where the orifice size is controlled in real time during the passage of two immiscible fluids, thereby allowing immediate variation of bubble sizes [106]. When vFF techniques are incorporated with an extrusion printer, 3D structures can be fabricated with varying yet controlled internal porous architecture as a result of bubble size variation. This is an example of coupling microfluidic liquid foam templating techniques with other methods. It has also been reported that microfluidics can also be incorporated with 3D bioprinting [104] and conventional techniques such as electrospinning [116].

Despite recent advances in solid foam fabrication from liquid templates, there are still challenges to be addressed, especially on the stability of the liquid template structure, ensuring that throughout the solidification process, the foam structure is preserved. Solidification entails drying procedures, which can have non-negligible influence on a foam’s morphology and its porous structure [103]. A detailed review on the stability of liquid foam templates as routes for solid foam fabrication can be found in [36].

## 4. Methods of Characterization of Liquid and Solid Foams

To evaluate the suitability of a foam for a specific application, the foam morphology, structure, and chemistry are the main properties to be analyzed [59,98].

Indeed, both liquid (Table 1) and solid foams (Table 2) can be described by morphological, structural, and chemical parameters, such as the average bubble or pore size of liquid or solid foams respectively, the liquid/solid fraction, the elastic modulus, or the surface chemistry.

The time evolution of a foam’s morphological characteristics gives insights about its stability. Liquid foams can be used, as they are or as templates in the production of well-defined porous solids through liquid templating, which in turn can find applications in the design of advanced materials with specific properties. In this latter case, the liquid foam stability plays an important role in guaranteeing the preserving of the structure during the templating of the foam. To study the stability of a foam (controlled by aging phenomena), liquid foams are collected in test tubes and pictures are taken just after their collection at different time intervals. The height of a foam is tracked with time, and the foam stability is determined by evaluating the decay of the liquid foam height [59]. Conventional microscopy (optical or confocal) can also be used to assess the stability of a foam. By recovering the sample on microscope slides, 2D and 3D foam images can be analyzed by using image analysis tools (such as Matlab or ImageJ) that detect bubbles, calculate their areas, and calculate their perimeters at different time steps. That way, the bubble size distribution is tracked with time, giving information about the foam aging phenomena.

Computer simulations and analytical calculations play an increasing role in morphological and structural characterizations of liquid and solid foams. For example, starting from experimental images of 2D and 3D foams, various aspects of foam behavior, such as coarsening, drainage [117], and mechanical behavior, can be simulated and compared with experiments using different software, such as the Surface Evolver (SE) [118,119,120].

To predict overall foam stability, surface-rheological properties of its liquid phase (aqueous surfactant solutions) can be also explored. A mechanical stress is applied to the interface by means of an oscillation or expansion of a drop, and the change of surface or interfacial tension is measured as a reaction to the applied mechanical stress.

Liquid and solid foams have distinctive mechanical or rheological properties qualifying them for many applications [61,71,121]. Literature about rheology of liquid foams is wide and complex [122,123]. They can have interesting elastic, plastic, or viscous properties, and most commonly, a combination of all. The elastic and viscous moduli, the yield stress, etc., can be measured using a rheometer, which involves shearing foams using different geometries (two planes; cone-plane; two cylinders) under controlled conditions (constant shear stress, shear rate, etc.) [124]. Tensile and compressive tests can be performed on solid foams by a texturometer or an electromechanic dynamometer [125].

For a solid foam, scanning electron microscopy (SEM) can be used in order to visualize the morphology and the pore size distribution [61]. Moreover, SEM combined with energy dispersive X-ray spectroscopy (EDX) can give information about the elemental chemical composition of the foam by mapping selected point locations on the sample at the same time. The EBSD (electron back scatter diffraction) technique can be used to characterize the texture of a foam and its local crystallographic orientation [126]. X-ray diffraction (XRD) and X-ray micro computed tomography (X-ray micro CT) are used to measure the porosity [127]. The density and the porosity values of the foams can also be estimated measuring the dimensions and the mass [62].

To characterize a foam from a chemical point of view, thermal analysis can be conducted. Different techniques can be used to characterize the foam materials when heated, cooled, or held isothermally. Differential scanning calorimetry (DSC) is used for measuring the heat flow properties [98]; thermogravimetric analysis (TGA) for the determination of the weight loss properties [71]; thermomechanical analysis (TMA) for the dimensional properties; dynamic mechanical analysis (DMA) for the characterization of the mechanical or viscoelastic properties; and gas permeation chromatography for quantifying the molecular weight distribution [21].

Starting from this panel of characterization tools (Table 1 and Table 2 for liquid and solid foams, respectively), specific characterization tests can be additionally performed depending on the application. As an example, in the automotive industry where foams are used to prevent injuries, they can be exposed to quasi-static and dynamic compression loading, to determine their energy absorption characteristics and impact behavior [128]. For sound insulation, acoustic properties can also be measured using ultrasound transmission [129]. If we look at the biomedical field, the main topic of this review, the use of foams for dermatology requires the investigation of the bubble size, the texture, the stability, and the rheological properties [13]. For a scaffold to be applied in tissue engineering, a thorough understanding of the chemistry and physicochemical properties of the tissue to be engineered and the materials used in the process are required. Indeed, the balance between basic requirements, including material biocompatibility (in vitro and in vivo studies) [71] and biodegradability [101], along with morphological (porosity, pore size, interconnectivity) and structural properties (e.g., compressive and tensile strength), is a key point to take into account [22]. From this perspective, the engineered scaffolds must exhibit tissue-like functional properties, including mechanical behavior comparable to the native tissues they have to substitute. In addition, the wettability of the scaffolds can be an important feature and is evaluated in each case by contact angle measurements [130]. The next section sheds light on the different biomedical applications of foams and scaffolds that require the previously mentioned features.

## 5. Foams for Biomedical Applications

Porous materials like foams are widely used in the biomedical field due to their interesting properties, such as lightweight structures, strong mechanical properties, porous networks, large surface areas, potentially controlled degradation, and biocompatibility.

The main biomedical applications of liquid foams include cosmetics and dermatology treatments, especially in the fabrication of foam baths, creams, etc. There has also been a growing number of foam products in the pharmaceutical market; that has attracted researchers due to the potential ability of pharmaceutical foams to enhance topical drug delivery and bioavailability [13]. Foams have also been used as delivery vehicles for peptides [131], drugs [7,8], or cells, and provided as innovative alternatives to creams and ointments [8,9]. In general, the release mechanism is very process-sensitive and can be controlled by encapsulation, tailored polymeric degradation, or attachment of signaling molecules to the polymer surfaces of the systems [17]. This control can be achieved by adopting microfluidics as a generation process since it allows controlled reactions between the different components. Indeed, if so far, microfluidics has still not been extensively explored to optimize the formulations of liquid foams for such applications, its potential to improve the controllability of materials characteristics has been confirmed by allowing tunable fabrication of other types of drug delivery carriers, such as polymeric particles [132,133].

Microfluidics has been more used to generate solid foam-based scaffolds for cell culture and tissue engineering. Tissue engineering is defined as “an interdisciplinary field that applies the principles of engineering and life sciences to the development of biological substitutes that restore, maintain, or improve tissue function or a whole organ” [134]. In general, tissue engineering comprises in vitro 3D cell culture and in vivo tissue-induced regeneration applications, and for both, the biological cross-talk between cells and the scaffolds is controlled by the properties of the materials and the characteristic design of the scaffold [135]. In fact, tissue engineering aims to restore damaged human native tissues through the use of biocompatible and biodegradable structures which become integrated into the body. In the last few years, this field has been advancing and exploring almost every tissue and organ of the human body [22]. In tissue engineering, cells are allowed to proliferate and organize their the extracellular matrix (ECM) in a three-dimensional scaffold to form, ex vivo, a clinically functional tissue, exhibiting histochemical, biochemical, and biomechanical properties identical to said native tissue [136]. Recently, 3D solid foam structures have been extensively designed as scaffolds for tissue engineering applications in order to mimic the properties and functions of ECM by providing structural support for cells to attach, grow, migrate, and differentiate [22].

By presenting an alternative to using in vivo animal models for testing, 3D cell culture systems are gradually replacing 2D ones, mainly because they represent a more realistic model of the human body. Indeed, studies of the effect of drug dosage in both media show that cells respond differently to drugs in 2D versus 3D, probably due to isotropy of cell growth in the latter ones [60]. Moreover, research has showed that the microenvironment around the cells can affect different mechanisms, such as drug responses and delivery [137]. 3D scaffolding is a critical component in tissue engineering because it provides the clues for cell seeding, migration, growth, and expansion towards new tissue formation [62]. As mentioned earlier, a scaffold’s excellent performance can depend not only on different factors such as the pore size [138], surface area, porous structure, or the degree of pore interconnectivity, but also on the choice of the materials used and their different fabrication processes. The ideal materials used for foam-based scaffold should be biodegradable and bioabsorbable to support the replacement of new tissues. The scaffolds should also be biocompatible to avoid inflammation reactions [101,139] and should also possess proper mechanical properties (for example, a defined elastic modulus, flexural modulus, tensile strength, and maximum strain) and degradation rate to support the growth of new tissues [22,140]. Natural materials (i.e., silk, collagen, chitosan, gelatin, hyaluronic acid, alginate, and others) are favored but can sometimes lack mechanical strength compared to the synthetic ones (PEG, PMMA, PGA, and PLGA), and thus, often, a combination of materials is used to combine the different advantages. In some cases, nanoparticles are also introduced into the scaffolds to improve the mechanical properties. From that perspective, an interesting study was proposed by Boccaccini et al. [101] in which they developed poly(D,L-Lactide) (PDLLA) foams with TiO${}_{2}$ nanoparticles and PDLLA/TiO${}_{2}$-bioglass foam composites for bone tissue engineering scaffolds [32,71,101].

Materials used in this context are in most cases of polymeric origin [30]. In recent years, biodegradable compressible foams based on a mixture of poly(lactic-co-glycolic Acid) (PLGA), polycaprolactone (PCL), and poly(L-lactide-co-ϵ-caprolactone) (PLCL) for negative pressure wound therapy have been fabricated [55]. Density graded polymer foams have also been explored for interfacial tissue engineering and showed interesting mechanical behavior [141]. Lo et al. [142] fabricated a variety of highly porous biodegradable polymer (poly-L-lactide, PLLA) foams for cell transplantation devices and tissue grafts. They demonstrated the ability for controlled delivery by studying the release of small hydrophobic and hydrophilic molecules from these highly porous structures. Hydrogels constitute another class of attractive materials as scaffolds for cell delivery and drug-screening applications thanks to their high water content, their similarity to the native ECM and biocompatibility, and their easily tunable mechanical, chemical, and physical properties [143]. Using these various materials, different kinds of porous structures can be developed for scaffolds in tissue engineering for various types of tissues [32]. Indeed, an excellent scaffold performance for tissue engineering lies in the strict tuning of different features and functionalities of the foams. In this case, the pore sizes and interconnection degree are crucial elements in controlling cell behavior and new tissue regeneration. Many studies have shown the influence of the scaffold pore characteristics (size, pore interconnectivity, and wettability) on cell culture behavior and drug delivery. For instance, Canal et al. [144] have reported macroporous solid foams generating a rough topography, giving the material superhydrophobic properties that affect lipophilic active principles in drug delivery. Moreover, depending on the scaffold pore size, different cell culture behaviors have been observed. For instance, macroporous foams (around hundreds of microns) have been proven to promote the in vitro cell invasion and differentiation, as well as the in vivo infiltration of the surrounding tissue, such as in the case of bone regeneration [71,145]. Furthermore, microporous foams (pore size ranging from one to 50 microns) promoted the transport of fluids to the cell and tissue [71,145]. As a whole, nano-pores play crucial roles in the formation of collagen fibers and ECM [146], whereas micro and macro-pores played an important role in cell seeding, distribution, and further biomechanical properties of cells.

Different studies explored the interconnectivity control and effect on the cell culture. In 2001, Ma et al. described the well-controlled interconnected macroporous structure of biodegradable PLLA and PLGA polymer scaffolds obtained through a novel solvent-casting technique [62]. Griffon et al. [136] studied the effect of pore interconnectivity on chondrocyte proliferation and function within chitosan-based scaffolds and compared the potential of chitosan and polyglycolic acid (PGA) matrices for chondrogenesis. They demonstrated that increasing pore interconnectivity of the chitosan scaffold resulted in the production of constructs with more chondrocytes and matrices, thereby improving the diffusion of nutrients and cells throughout the scaffold.

The control over a scaffold’s porous structure is process-dependent. The advancement of rapid prototyping techniques can significantly improve the regulation of the pore network, thereby playing a role on cell seeding and culturing [147]. From another perspective, the literature related to the fabrication of foams as scaffolds through conventional gas foaming is quite large [61,101], even though these conventional methods suffer from several limitations when cell culture comes into play. It has been shown, for example, that the cell suspension follows preferential flow paths across the scaffold when cells are seeded under perfusion on a gas-foamed scaffold. Furthermore, due to a discontinuous permeability within the scaffold, cell proliferation, differentiation, and migration are site dependent, causing the premature failure of scaffolds within in vivo applications [147]. All-in-all, the conventional techniques do not meet the full requirements encountered in applications, such as uniformity, portability, and quantity.

Alternatively, foam fabrication using microfluidics supports precisely controlled mixing of reagents and components, offering the possibility to design tailored scaffolds with tunable pore characteristics [17,29,111]. In 2015, Costantini et al. [38] investigated the link between polymer solution properties and the scaffold’s porous characteristics. The pore interconnectivity level was tuned through the variation of surfactant concentration. Consequently, this affects the permeability of the materials, a factor of key importance in flow-through applications in tissue engineering. Using microfluidics, Elsing et al. [148] achieved introducing a defined pore size gradient in polymer foams using foamed emulsions as templates. Recently, Andrieux et al. [59] presented a microfluidic flow-focusing study to produce biopolymer foams with tunable pore size distributions (mono- versus polydisperse) and different pore organizations (ordered versus disordered). Microfluidic mediated foams applied to tissue engineering have gained increasing interest over the years due to the described control of pore interconnectivity, which often results in pore uniformity. Mei et al. [149] reported microfluidic based 3D cell culture scaffolds used to repair cardiac tissue. Type I collagen and gelatin, two of the ECM molecules, were selected as the scaffolding materials for cell seeding. Owing to a flow focusing microfluidic device, scaffolds with variable pore sizes can be designed to adapt to various needs of different cell types. Colosi et al. [108] have produced poly(vinyl alcohol) (PVA) foams by exploiting and comparing two different production techniques: a microfluidic foaming technique and a traditional gas foaming technique. Results showed that the microfluidic produced scaffold generated a much more uniform porous texture than the gas-foaming one as witnessed by narrower pore size, interconnection, and wall thickness distributions. On the other hand, limited production rate represents the principal disadvantage of microfluidic foaming as a scaffold fabrication method, emphasizing the ongoing need to improve this technique.

## 6. Conclusions

Numerous examples of foam-based scaffolds used in the biomedical field have been fabricated using conventional means. While many would consider these conventional means “tried-and-tested”, they do still present glaring disadvantages. However, researchers have now increasingly recognized the potential of using microfluidics to fabricate these foam scaffolds due to undeniable advantages, such as control of porous structures. A growing number of papers cited in this review have reported different techniques to improve control and design of a foam’s porous structure, not just in terms of morphology but also in terms of its mechanical properties (stability, elasticity, etc.). In fact, the literature is abound with different techniques on microfluidics-based foam production [90,109,111,150]. Indeed, there has been significant progress towards the development of foams with different properties using microfluidics for a wide variety of biomedical applications, including drug delivery. This is due to its advantages such as cost, ease of parallelization, and highly controlled production; and precise tuning of pore structure design, morphology, and interconnectivity. Pore structures with defined orders have been shown to influence cell interactions, which in turn can affect cell seeding and distribution, and the release and delivery of molecules or active principles. In this regard, microfluidics-mediated foam production is rapidly being recognized as an alternative yet advanced tool to fabricate well-defined scaffolds for tissue engineering and drug release applications.

## Figures and Tables

**Table 1:** Methods of characterization of liquid foams.

		Characteristics	Tools
**LIQUID FOAMS**	**Morphology** **(foam architecture)**	Bubble size	Photographs/images
		Porosity	SEM
		Liquid Fraction	Confocal microscopy
		Electrical conductivity	Optical microscopy
		Interconnectivity	X-ray radioscopy
		Ageing phenomena	Conductivity meter
			Simulations (e.g., Surface Evolver)
	**Mechanical Behavior** **(foam performance)**	Surface Tension	Tensiometer
		Viscosity	Viscometer
		Elastic modulus	Rheometer
		Viscous Modulus	Simulations
		Yield Stress	
	**Chemistry** **(foam composition)**	Thermal Analysis	DLS
		Surface Energy	TGA
		Chemistry charge	TMA
		Interface adherence	

**Table 2:** Methods of characterization of solid foams.

		Characteristics	Tools
**SOLID FOAMS**	**Morphology** **(foam architecture)**		Photographs/images
			SEM/TEM
		Pore size	Confocal microscopy
		Porosity	Optical microscopy
		Microstructure	X-ray diffraction
		Electrical conductivity	Conductivity meter
		Interconnectivity	Simulations (e.g., Surface Evolver)
			X-ray Micro-CT
			EBSD
	**Mechanical Behavior** **(foam performance)**	Elastic modulus	
		Flexural modulus	Mechanical tests
		Compressive Strength	DMA
		Tensile Strength	Surface Evolver
		Yield Stress	
	**Chemistry** **(foam composition)**	Thermal Analysis	DLS
		Surface Energy	TGA
		Chemistry charge	TMA
			SEM-EDX
